# Wearable Activity Trackers and Physical Activity Levels Among Members of the Athens Medical Association in Greece

**DOI:** 10.3390/jcdd11100336

**Published:** 2024-10-21

**Authors:** Stamatios Lampsas, Georgios Marinos, Dimitrios Lamprinos, Panagiotis Theofilis, George E. Zakynthinos, Ioannis Gialamas, Antonios Lysandrou, Sotirios Pililis, Loukia Pliouta, Georgia Tzioumi, Eleni Anastasopoulou, Vaia Lambadiari, Evangelos Oikonomou, Gerasimos Siasos

**Affiliations:** 1 3rd Department of Cardiology, Thoracic Diseases General Hospital Sotiria, Medical School, National and Kapodistrian University of Athens, 11527 Athens, Greece; lampsas.stam@gmail.com (S.L.);; 2 Department of Hygiene, Epidemiology and Medical Statistics, Medical School, National and Kapodistrian University of Athens, 11527 Athens, Greece; gmarino@med.uoa.gr (G.M.); dimitrislamprinos@gmail.com (D.L.); 3 1st Department of Cardiology, Hippokration General Hospital, Medical School, National and Kapodistrian University of Athens, 11527 Athens, Greece; 4 2nd Department of Internal Medicine, Attikon University Hospital, Medical School, National and Kapodistrian University of Athens, 12462 Athens, Greece; sotiris181@yahoo.gr (S.P.);; 5 Cardiovascular Division, Harvard Medical School, Brigham and Women’s Hospital, Boston, MA 02115, USA

**Keywords:** exercise, wearable activity trackers, doctors, physicians, physical activity

## Abstract

Introduction: Wearable Activity Trackers (WATs) offer real-time feedback on activity levels. We assessed the impact of WAT usage on physicians’ exercise habits. Methods: Physicians from the Athens Medical Association, Greece (n = 742) responded to a self-administered questionnaire evaluating usage of WAT, demographic characteristics, specialty, and physical exercise habits. WHO guidelines recommend at least 150 min/week of moderate-intensity exercise in all healthy adults. Subjects were divided in Users of WATs (Group A), and Non-Users of WATs (Group B). This is an observational, cross-sectional study. Results: There was no difference in baseline characteristics between the two groups (age, sex, body mass index). WATs were used by 38%. Between Group A and B, there was difference in mean exercise training time (302 ± 304 min vs. 210 ± 268 min, *p* < 0.001), higher percentage of WHO goal achievement (66.3% vs. 50.7%, *p* < 0.001), and greater awareness of WHO Guidelines (59.9% vs. 47.4%, *p* < 0.001). WATs were mostly used by four main specialties, with higher use from Cardiologists: Cardiology (47%), Endocrinology (44%), Surgery (35%) and Internal Medicine (25%), with a *p* = 0.045. Finally, users of WATs compared to non-users showed higher willingness to reduce body weight (58.5% vs. 48%, *p* = 0.01), apply dietary restrictions (36.5% vs. 29.6%, *p* = 0.05), and greater motivation for weekly physical exercise (74.1% vs. 32.4%, *p* < 0.001); Conclusion: Physicians using WATs demonstrate increased exercise training time, greater awareness of WHO guidelines and a higher propensity to implement dietary restrictions compared to non-users. Variations in WAT usage across medical specialties emphasize the need for targeted interventions to promote physical activity and enhance healthcare professionals’ health.

## 1. Introduction

Ancient Greek physicians, including Hippocrates in around 500 BC, recognized the importance of physical exercise for improving overall health and performance. Hippocrates stated, ‘All parts of the body which have a function, if used in moderation and exercised in labors to which each is accustomed, become healthy, well-developed, and age more slowly, but if unused they become prone to disease, defective in growth, and age quickly’ [1]. Similarly, Plato emphasized the significance of physical activity, asserting that ‘Lack of activity destroys the good condition of every human being, while movement and methodical physical exercise preserve and enhance it.’ Nevertheless, in the early 1900s, patients with acute myocardial infarction were often advised to remain on complete bed rest [2]. Insufficient physical activity poses a significant threat to global health, due to modern conveniences, like labor-saving devices and transportation advancements [3]. The latest global data indicate that about one in four adults (27.5%) and over three-quarters of adolescents (81%) fail to meet the recommended guidelines for aerobic physical activity [4,5]. Physical inactivity significantly contributes to premature mortality and morbidity, increasing the risk for more than 25 chronic medical conditions [6]. Globally, physical inactivity is estimated to be responsible for 7.2% of all-cause deaths and 7.6% of deaths due to cardiovascular disease (CVD) [7,8].

Since 1953, when the first epidemiological study investigated the role of physical activity in chronic disease risk [9], the impact of exercise has been debatable. Nowadays, physical activity provides strong evidence for reducing cardiovascular mortality, all cause atherosclerotic coronary heart disease, risk of ischemic stroke, prevalence of type 2 diabetes mellitus and improving mental health, such as depression, chronic anxiety and panic disorders [10,11,12,13,14]. The latest World Health Organization (WHO) guidelines highlight the enduring relevance of Hippocrates’ and Plato’s ideas, advocating for people of all ages and abilities to participate in regular physical activity [3]. The WHO recommends that adults engage in at least 150 to 300 min of moderate-intensity aerobic physical activity, or 75 to 150 min of vigorous-intensity aerobic physical activity, per week, or an equivalent combination of both [3]. In recent decades, lifestyle interventions, including self-monitoring behaviors, exercise prescriptions and personalized counseling, have proven effective at increasing physical activity levels in the short term [15]. Wearable activity trackers are consumer-based wearable devices allowing individuals to objectively monitor their physical activity levels, which have gradually become part of daily life, attracting a large user base due to their affordability [16]. In addition, this technology offers an alternative way of providing continuous support and motivation for individuals aiming to either increase their activity levels or maintain them after a structured lifestyle intervention [17].

Nowadays, physicians deal with extremely concerning levels of stress, psychological and physical exhaustion. According to a large study, 45% of physicians had experienced at least one symptom of burnout, while a large online survey revealed that 39.8% of physicians had suffered from burnout in the past [18,19]. Physicians’ exercise habits significantly impact their patients’ treatment and their daily decision-making performance, since their effectiveness and counseling performance is highly dependent on their overall mental and physical condition [20]. Despite this, there are limited data on the impact of wearable activity trackers on physicians’ lifestyle and on their professional performance. The aim of this cross-sectional study is to assess how their everyday habits are affected by the usage of these devices and to evaluate the adoption and achievement of physical activity goals among different medical specialties within the members of the Athens Medical Association (AMA), Greece.

## 2. Materials and Methods

### 2.1. Study Design

This is a cross-sectional, observational, descriptive study. This survey took place from October 2023 to January 2024. An anonymous self-administered questionnaire was disseminated online among the members of the Athens Medical Association (AMA). The inclusion criteria were as follows: 1. active membership in the AMA; 2. access to the internet; 3. voluntary participation of physicians based in Athens. Participants were informed to consent before participation. The investigation conforms to the principles outlined in the Declaration of Helsinki [21]. The study protocol was approved by the AMA’s Ethical Board (protocol number: 13-07-2023).

The questionnaire included questions on the socio-demographic background of the participants (age, sex, weight, height, medical history, medical specialty and occupational characteristics). In addition, the questionnaire contained questions on their participation in 24-h overnight shifts (“Do you participate in 24-h overnight shifts?”; “If yes, how many shifts do you have per month?”), to evaluate if these shifts affect their physical activity habits. Moreover, close-ended questions regarding their dietary habits were included (“Do you make any effort to lose body weight?”; “Do you apply dietary restrictions to reduce body weight?”) and questions about the significance of WATs on their exercise habits (“Do you believe that the daily usage of Wearable Activity Trackers motivated you to do regularly physical exercise?”). Finally, to evaluate physicians’ weekly physical activity habits, questions regarding the duration of their activities (“How many minutes of moderate-intensity exercise do you have per week?”), the type of exercise they prefer (“What type of exercise do you have?”), and their awareness of Guidelines on physical activity (“Do you know which is the recommended duration of weekly moderate-intensity exercise according to the World Health Organization?”) were included. Participants were categorized into two groups based on their regular use of WATs. Group A consisted of those who have used WATs daily for more than one year, while Group B included participants who do not use WATs on a regular basis.

### 2.2. Wearable Activity Trackers and Physical Activity

Participants were asked whether they have used wrist-wearable trackers during the past 12 months (“Do you use wrist-wearable activity trackers to evaluate your everyday physical activity habits and daily steps, e.g., smartwatches?”). Physical activity was evaluated according to the World Health Organization (WHO) 2020 Guidelines on Physical Activity, which is based on objective measurements [3,22]. In particular, all adults of all age groups should undertake at least 150 min per week (min/week) of moderate-intensity exercise (ΜΙΕ), or 75 min/week of vigorous-intensity exercise (VIE) [3]. An equivalent combination of moderate-intensity and vigorous-intensity physical activity was calculated as follows: TOTAL min/week = (MIE) + 2 × (VIE). Participants who did more than 150 min of moderate-intensity exercise per week achieved the goal aimed for in the WHO Guidelines on Physical Activity.

### 2.3. Statistical Analysis

The statistical analyses were conducted using IBM SPSS Statistics software (IBM Corp. Released 2021. IBM SPSS Statistics for Windows, Version 28.0. Armonk, NY, USA: IBM Corp). Continuous variables were assessed for normality using the Kolmogorov–Smirnov test and P–P plots. Normally distributed variables are expressed as mean ± standard deviation. Categorical variables are reported as frequencies and percentages. Differences between categorical variables were evaluated using contingency tables and χ^2^-tests, while differences in continuous variables were assessed using independent t-tests. All calculations were based on two-sided tests. Statistical significance was set at *p*-values < 0.05.

## 3. Results

### 3.1. Study Population

Out of 22,000 members of the AMA who were registered to the newsletter list, 768 responses were received (response rate: 3.5%). The study population consisted of 742 participants after twenty-six responses excluded due to incomplete data. The mean age was 50.1 ± 12.8 years, and the average body mass index (BMI) was 25.4 ± 4.3 kg/m^2^, with 51.2% self-characterized as males. The prevalence of smoking among the participants was 20.2%, 4.6% of the participants reported having cardiovascular disease, and 9.0% had a history of diabetes mellitus.

Participants were classified into four medical specialty divisions according to their medical specialty. Particularly, the majority of the participants were categorized in the broad category Internal Medicine (55.9%, n = 415); a large proportion were Surgeons (31.9%, n = 237); a smaller proportion of 9.9% (n = 66) were physicians in the Diagnostics and Laboratory field, and only a few participants were in the Mental Health division (3.2%, n = 24). Specifically, the medical specialties with the most physicians were as follows: 1. Internal Medicine: 9.6% (n = 71); 2. Cardiology: 9.2% (n = 68); 3. Pediatrics: 8.5% (n = 63); 4. General Surgery: 4.9% (n = 36). Furthermore, most of the participants did not participate in 24-h shifts 60.4% (n = 448), and the rest (39.6%, n = 294) did at least one 24-h shift per month. Of the subjects that participated in shifts, 21.4% (n = 159) did more than five 24-h shifts per month, and 18.2% (n = 135) did 1–4 24-h shifts per month.

### 3.2. Physicians’ Exercise Habits and WATs

A considerably high proportion of the total population uses WATs daily 38% (n = 282) (Table 1). There was no difference in baseline characteristics between Group A and Group B, respectively: age (49.1 ± 12.2 vs. 50.8 ± 13.1 years, *p* = 0.07), male sex prevalence (55.3% vs. 48.7%, *p* = 0.08), BMI (25.8 ± 4.7 vs. 25.3 ± 4.1 kg/m^2^, *p* = 0.11), smoking (18.9% vs. 21.1%, p=0.46), Diabetes Mellitus prevalence (7.8% vs 9.8%, *p* = 0.36), and cardiovascular disease prevalence (5.3% vs. 4.1%, *p* = 0.45).

**Figure 1:**
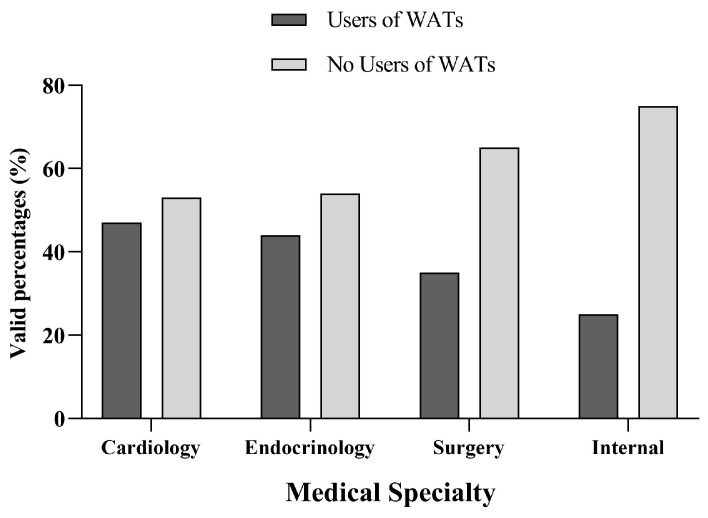
Usage of wearable activity trackers (WATs) among different medical specialists in the Athens Medical Association (AMA).

WATs were mostly used by 4 main specialties: Cardiology (47%), Endocrinology (44%), Surgery (35%), and Internal Medicine (25%), with cardiologists reporting the higher usage (*p* = 0.045) (Figure 1).

Moreover, users of WATs (Group A) compared to non-users (Group B) showed a higher participation rate in 24-h shifts per month (44.7% vs. 36.5%, *p* = 0.02). Group A when compared to Group B also reported a higher willingness to reduce body weight (58.5% vs. 48%, *p* = 0.01), applied dietary restrictions in order to lose body weight (36.5% vs. 29.6%, *p* = 0.05), and supported that the usage of WATs tends to motivate them in weekly physical habits (74.1% vs. 32.4%, *p* < 0.001).

**Figure 2:**
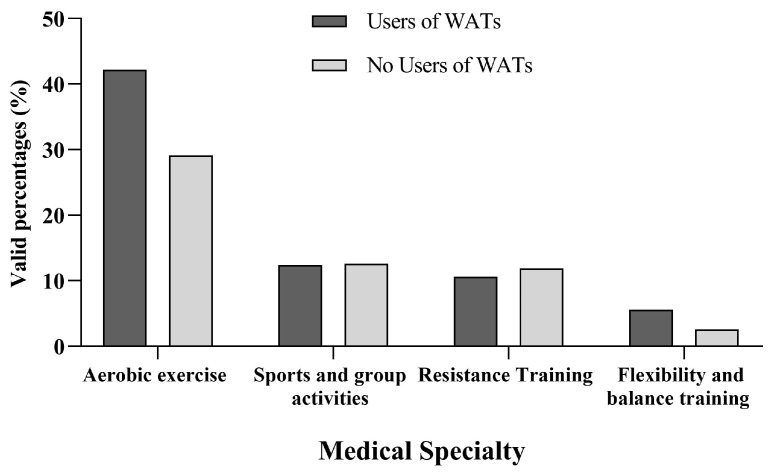
Different types of physical activity exercise and the usage of wearable activity trackers (WATs) among physicians in the Athens Medical Association (AMA).

Interestingly, we observed that users of WATs (Group A) compared to subjects without WATs (Group B) reported a higher percentage of regular weekly exercise, respectively (70.9% vs. 56.3%, <0.001), with the majority preferring aerobic exercise (42.2% vs. 29.1%, *p* = 0.01) (Figure 2). Notably, there was a difference in mean exercise endurance training time between Group A and Group B (302 ± 304 min vs. 210 ± 268 min, *p* < 0.001). A higher percentage of participants from Group A compared to Group B followed the Guidelines for physical activity (66.3% vs. 50.7%, *p* < 0.001). Awareness of the Guidelines on Physical Activity was (59.9% vs. 47.4%, *p* < 0.001) for Group A and respectively. Moreover, specialties that reported the highest awareness of ESC Guidelines were Cardiology (88%), Internal Medicine (64%), and Pediatrics (60%), with a *p* < 0.01.

## 4. Discussion

This study examined the use of WATs and the levels of physical activity exercise among Athens Medical Association (AMA) members in Greece. The findings revealed that a significant proportion of the physicians (38%) use WATs on a daily basis. Moreover, insights showed that WAT adoption among physicians is linked to higher physical activity levels, awareness of physical activity guidelines, and adoption of healthy lifestyle behaviors.

Notably, this study showed variations among different medical specialties in WAT usage, since Cardiologists, Endocrinologists, Surgeons, and Internists showed the highest adoption rates, with Cardiologists reporting the highest (47%) percentage of WAT usage.

The literature supports that these state-of-the-art devices are correlated with lower cardiovascular disease mortality risk related to sitting time [23], and their usage in patients with heart failure for self-monitoring and motivational purposes is associated with increased levels of physical activity and is predictive of disease severity [24]. Although the clinical integration of wearable devices remains in the earliest stages, they can facilitate lifestyle changes for primary prevention, enable arrhythmia screening and support the remote management of patients with existing conditions, such as heart failure or peripheral artery disease [25]. This study also suggested that WATs are effective tools for increasing physical activity levels among physicians, potentially by providing real-time feedback and fostering self-monitoring behaviors, since WAT users showed higher rates of achievement related to the WHO Guidelines on physical activity [3].

In addition, the study investigated the role of WATs in physical activity behaviors, as a substantial proportion of WATs users (74.1%) reported that these devices motivated them to engage in regular physical exercise, compared to only 32.4% of non-users. Moreover, users of these devices showed a greater inclination toward dietary modifications, which can be combined with diet-tracking apps or features that allow users to monitor both caloric intake and expenditure. Interestingly, despite the impact of occupational factors, such as participation in 24-h shifts, a higher percentage of WAT users participated in 24-h shifts suggesting that, even with demanding work schedules, WAT users were more likely to engage in physical activity. Even though in the general population over 80% of adults remain insufficiently active, WATs were shown to encourage physical activity along with higher knowledge of physical activity guidelines, leading to more informed decision-making regarding personal health and patient counseling [3].

Limitations of this study include its cross-sectional design. which limits its ability to establish causal relationships. Moreover, the survey is based on self-reported data that may introduce biases. such as recall bias or overreporting of physical activity levels. and lacks objective measurements through direct observation. Furthermore, the response rate was relatively low (3.5%), which may limit the generalizability of the findings. Another key limitation of this study are the lack of detailed data on the specific types and models of WATs used by participants. Future research should focus on objective data from WATs and their effect on psychological, occupational, and specialty-specific factors. providing deeper insights into their effectiveness.

## 5. Conclusions

This study underlines the potential benefits of WATs’ enhancement of physical activity levels among physicians who, despite their busy schedules and high-stress occupations, can benefit from these devices in strengthening their health and well-being. WATs also facilitate greater adherence to recommended Guidelines. but also serve as valuable tools for motivation and lifestyle management. The findings advocate that these devices can contribute to broader public health improvements. not only for healthcare providers but also for a wide range of diseases and patients.

## Figures and Tables

**Table 1:** Physicians’ exercise habits, depending on the usage of Wearable Activity Trackers (WATs) or not.

	Users of WATs (Group A)	No Users of WATs (Group B)	*p*-Value
Subjects, % (n)	38 (282)	62 (460)	
Age, years	49.1 ± 12.2	50.8 ± 13.1	0.07
Male sex, % (n)	55.3 (156)	48.7 (224)	0.08
BMI, kg/m^2^	25.8 ± 4.7	25.3 ± 4.1	0.11
Smoking, % (n)	18.9 (53)	21.1 (97)	0.46
Diabetes Mellitus, % (n)	7.8 (22)	9.8 (45)	0.36
Cardiovascular Disease, % (n)	5.3 (15)	4.1 (19)	0.45
Participation on 24-h shifts, % (n)			
None, % (n)	55.3 (156)	63.5 (292)	0.04
Moderate (1–4 24-h shifts per month), % (n)	18.8 (53)	17.8 (82)	
High (≥5 24-h shifts per month), % (n)	25.9 (73)	18.7 (86)	
Effort to reduce body weight, % (n)	58.5 (165)	48 (221)	0.01
Dietary restrictions to reduce body weight, % (n)	36.5 (103)	29.6 (136)	0.05
Belief that the usage of wearable activity trackers motivates them, % (n)	74.1 (209)	32.4 (149)	<0.001
Type of weekly regular exercise, % (n)			
Aerobic exercise (e.g., running, swimming etc.), % (n)	42.2 (119)	29.1 (134)	0.01
Sports and group activities (football, basketball, tennis etc.), % (n)	12.4 (35)	12.6 (58)	
Resistance Training (e.g., weightlifting, body weight etc.), % (n)	10.6 (30)	11.9 (55)	
Flexibility and balance training (yoga, Pilates etc.), % (n)	5.6 (16)	2.6 (12)	
Minutes of moderate-intensity endurance exercise training per week (min)	302 ± 304	210 ± 265	<0.001
Achieve the goal of Guidelines on physical activity, % (n)	66.3 (187)	50.7 (233)	<0.001
Awareness for Guidelines on physical activity, % (n)	59.9 (169)	47.4 (218)	<0.001

Categorical variables are presented as valid percentages and continuous variables as mean ± standard deviation. BMI: Body mass index; min: minutes; WATs: Wearable Activity Trackers.

## Data Availability

The study data are available from the corresponding author on reasonable request.
